# Miscellany

**Published:** 1904-03

**Authors:** 


### Miscellany.

The Abuse of the Gold Cap.—One of the evils—growing evils, we may say—which needs persistent words of condemnation and censure is the abuse of the gold cap. Its easy adaptation renders it a too common product of "Dental Parlors," also of offices that have a professional standing but are sadly deficient in what is artistic.

It is almost never permissible to cover any of the ten anterior teeth, particularly of our lady patients, with one of these glaring abominations. It is a pitiful acknowledgment of inefficiency on the part of the operator who makes such use of them.

What is more distressing than the sight of a few gold caps to mar the smile and weaken the features of a beautiful and cultured woman? And yet this is a sight not infrequently met with. This disfigurement is conspicuous oftentimes with public speakers and persons whose taste should rebel against anything so unsightly.

In this day of porcelain art, when nature can be so closely imitated as to almost defy detection, such inartistic displays of dentistry have no excuse for being. One can scarcely attend a convention without encountering among the exhibitors from one to five outfits, all "superior methods" of swaging seamless gold crowns. The spirit of commercialism is here taking precedence over the artistic.

We are taught, and we should practise, the "art" of dentistry. And this means the restoration and repair of lost and injured members in such a manner that the casual glance will detect as little as possible of the inharmonious or artificial.—N. A. Stanley, D.M.D., New Bedford, Mass.

---

Grind Lower Teeth rather than force down.—Dr. J. N. Farrar says it is better to grind off the front lower teeth when too long rather than to attempt to crowd them down into their sockets. He grinds a little at a time at intervals of several weeks.

---

Reduction of Tendency to Hemorrhage.—Dr. C. Edward Wallis reports that ten grains of calcium chloride administered internally three times a day for a week previous to extracting reduces very materially the tendency to hemorrhage in patients who exhibit a predisposition to excessive hemorrhage.—*Dental Digest.*

---

To remove Oxide from German Silver.—Dr. V. E. Barnes, in the *Dental Summary,* suggests hydrochloric acid, full strength, used cold, to remove oxide from German silver regulating appliances.

---

Adrenalin: A New Hæmostatic.—This comparatively new drug is recommended for controlling hemorrhage in tooth extraction for patients with the hemorrhagic diathesis. Immediately before the operation a few drops injected into the gum around the tooth makes the extraction practically bloodless. It is also recommended as a preventive of the annoying hemorrhage attending the preparation of teeth or roots for crowning, and preparing for filling cavities which extend below the gum margin.—*Dental Cosmos,* May, 1903, page 399.

